# Brasilixanthone^1^

**DOI:** 10.1107/S1600536810027285

**Published:** 2010-07-21

**Authors:** Suchada Chantrapromma, Nawong Boonnak, Hoong-Kun Fun, Chatchanok Karalai, Kan Chantrapromma

**Affiliations:** a Crystal Materials Research Unit, Department of Chemistry, Faculty of Science, Prince of Songkla University, Hat-Yai, Songkhla 90112, Thailand; b X-ray Crystallography Unit, School of Physics, Universiti Sains Malaysia, 11800 USM, Penang, Malaysia; c Utilization of Natural Products Research Unit, Walailak University, Thasala, Nakhon Si Thammarat 80160, Thailand

## Abstract

The title xanthone [systematic name: 5,13-dihy­droxy-3,3,10,10-tetra­methyl-3*H*-dipyrano[3,2-*a*:2′,3′-*i*]xanthen-14(10*H*)-one], C_23_H_20_O_6_, was isolated from the roots of *Cratoxylum formosum* ssp. *pruniflorum*. There are two mol­ecules (*A* and *B*) in the asymmetric unit, which show chemical but not crystallographic inversion symmetry. The xanthone skeleton in both mol­ecules is approximately planar, with an r.m.s. deviation of 0.0326 (9) Å for mol­ecule *A* and 0.0355 (9) Å for mol­ecule *B* from the plane through the 14 non-H atoms. The pyran rings in both mol­ecules adopt sofa conformations. Intra­molecular O—H⋯O hydrogen bonds generate *S*(5) and *S*(6) ring motifs. Viewed onto the *bc* plane, the crystal structure resembles a herringbone pattern. Stacks of mol­ecules are stabilized by π–π inter­actions with centroid–centroid distances of 3.600 (5) Å. The crystal structure is further stabilized by weak C—H⋯O and C—H⋯π inter­actions.

## Related literature

For details of hydrogen-bond motifs, see: Bernstein *et al.* (1995 ▶) and for ring conformations, see: Cremer & Pople (1975 ▶). For background to xanthones and their biological activities, see: Boonnak *et al.* (2006 ▶, 2007 ▶, 2009 ▶); Hay *et al.* (2008 ▶); Mahabusarakum *et al.* (1983 ▶); Marques *et al.* (2000 ▶); Molinar-Toribio *et al.* (2006 ▶); Phongpaichit *et al.* (1994 ▶); Yu *et al.* (2007 ▶). For a related structure, see: Fun *et al.* (2006 ▶). For the stability of the temperature controller used in the data collection, see Cosier & Glazer (1986 ▶).
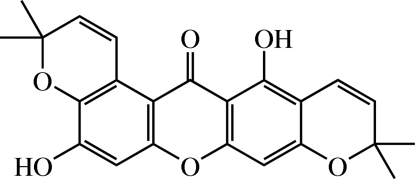


## Experimental

### 

#### Crystal data

C_23_H_20_O_6_
*M* *_r_* = 392.39
Monoclinic, 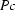

*a* = 7.5842 (3) Å
*b* = 12.2937 (4) Å
*c* = 19.6023 (6) Å
β = 96.827 (2)°
*V* = 1814.72 (11) Å^3^
*Z* = 4
Mo *K*α radiation
μ = 0.10 mm^−1^
*T* = 100 K
0.37 × 0.13 × 0.07 mm

#### Data collection

Bruker APEXII CCD area-detector diffractometer
Absorption correction: multi-scan (*SADABS*; Bruker, 2005 ▶) *T*_min_ = 0.963, *T*_max_ = 0.993
33718 measured reflections
3554 independent reflections
2954 reflections with *I* > 2σ(*I*)
*R*_int_ = 0.077

#### Refinement

*R*[*F* ^2^ > 2σ(*F* ^2^)] = 0.103
*wR*(*F* ^2^) = 0.270
*S* = 1.04
3554 reflections
453 parameters
2 restraints
H-atom parameters constrained
Δρ_max_ = 1.21 e Å^−3^
Δρ_min_ = −0.39 e Å^−3^

### 

Data collection: *APEX2* (Bruker, 2005 ▶); cell refinement: *SAINT* (Bruker, 2005 ▶); data reduction: *SAINT*; program(s) used to solve structure: *SHELXTL* (Sheldrick, 2008 ▶); program(s) used to refine structure: *SHELXTL*; molecular graphics: *SHELXTL*; software used to prepare material for publication: *SHELXTL* and *PLATON* (Spek, 2009 ▶).

## Supplementary Material

Crystal structure: contains datablocks global, I. DOI: 10.1107/S1600536810027285/bt5295sup1.cif

Structure factors: contains datablocks I. DOI: 10.1107/S1600536810027285/bt5295Isup2.hkl

Additional supplementary materials:  crystallographic information; 3D view; checkCIF report

## Figures and Tables

**Table 1:** Hydrogen-bond geometry (Å, °) *Cg*5 and *Cg*20 are the centroids of C5*A*–C8*A*/C12*A*/C13*A* and C5*B*–C8*B*/C12*B*/C13*B* rings, respectively.

*D*—H⋯*A*	*D*—H	H⋯*A*	*D*⋯*A*	*D*—H⋯*A*
O3*A*—H3*AA*⋯O2*A*	0.82	1.84	2.571 (9)	147
O5*A*—H5*AA*⋯O4*A*	0.82	2.19	2.656 (9)	116
O3*B*—H3*BA*⋯O2*B*	0.82	1.84	2.559 (9)	146
O5*B*—H5*BA*⋯O4*B*	0.82	2.15	2.628 (8)	117
C15*B*—H15*B*⋯O2*A*^i^	0.93	2.60	3.514 (11)	168
C16*A*—H16*A*⋯O2*A*	0.93	2.29	2.879 (11)	121
C16*B*—H16*B*⋯O2*B*	0.93	2.31	2.890 (10)	120
C17*B*—H17*E*⋯O5*B*^i^	0.96	2.58	3.441 (10)	149
C23*B*—H23*D*⋯O1*A*^ii^	0.96	2.59	3.370 (11)	139
C18*A*—H18*B*⋯*Cg*20	0.96	2.75	3.611 (10)	150
C18*B*—H18*D*⋯*Cg*5	0.96	2.79	3.662 (9)	152

Symmetry codes: (i) 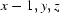; (ii) 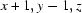.

**Fig. 1.**
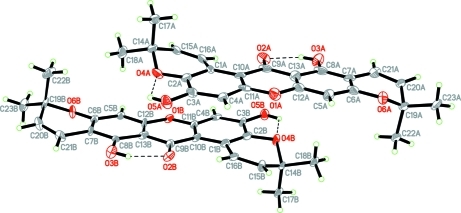
The structure of (I), showing 50% probability displacement ellipsoids and the atom-numbering scheme. Intramolecular O—H···O hydrogen bonds are shown as dashed lines.

**Fig. 2.**
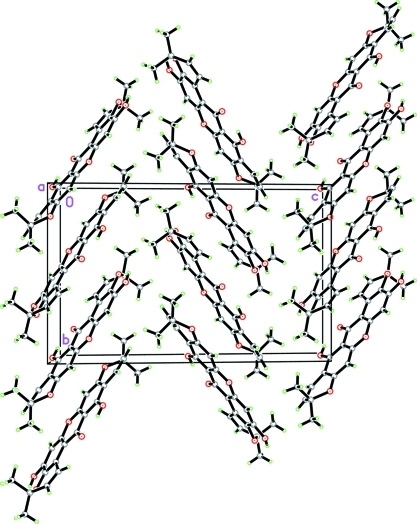
The crystal packing of (I) viewed along the a axis, showing zig-zag chains stacked along the b axis.

## supplementary crystallographic information

### Comment

Xanthones are secondary metabolites extracted from several plants and have demonstrated to possess considerable biological properties such as antibacterial, antioxidant, antiprotozoal and cytotoxic activities (Boonnak *et al.*, 2006; 2007; 2009; Mahabusarakum *et al.*, 1983; Molinar-Toribio *et al.*, 2006; Phongpaichit *et al.*, 1994; Yu *et al.*, 2007). During the course of our investigation of the chemical constituents and bioactive compounds from the *Cratoxylum formosum* ssp. *pruniflorum*, a Thai medicinal plant, the title xanthone (I) namely brasilixanthone (Marques *et al.*, 2000) was isolated from the roots of this plant. It was tested against fungi (*Candida albicans*) and both ordinary and antibiotic-resistant bacterial strains such as *Bacillus subtilis*, *Staphylococcus aureus* TISTR517, *Enterococcus faecalis* TISTR459, Methicillin-Resistant *Staphylococcus aureus* (MRSA) ATCC43300, Vancomycin-Risistant *Enterococcus faecalis* (VRE) ATCC 51299, *Salmonella typhi*, *Shigella sonei* and *Pseudomonas aeruginosa*. Our results showed that (I) does not possess antifungal and antibacterial activities against the tested pathogens with the MIC (Minimum Inhibition Concentration) > 300 µg/mol. Herein we report the crystal structure of (I).

Compound (I) crystallizes with two independent molecules (*A* and *B*) per asymmetric (Fig. 1). The conformations of molecule *A* differ from those observed in molecule *B* in which the two chromene rings in *A* pucker in the opposite direction from those in *B* (Fig. 1). In both molecules, the three ring system [C1–C13/O1] are essentially planar with the *r.m.s*. deviation of 0.0326 (9) and 0.0355 (9) Å, respectively for *A* and *B* from the plane through all 14 non-hydrogen atoms of the three rings and with a maximum deviation of -0.085 (9) Å (for *A*) and +0.081 (9) Å (for *B*) for atom C3. The O3 and O5 hydroxyl O atoms lie close to this plane with deviations +0.003 (6) for O3 and +0.023 (6) Å for O5 (in *A*) [the corresponding values are -0.018 (8) and -0.030 (1) Å in *B*]. The two chromene rings in *A*, (C1A-C2A/C14A–C16A/O4A; angular fashion chromene) and (C6A-C7A/C19A–C21A/O6A; linear fashion chromene), adopt screw-boat conformations (Cremer & Pople, 1975) with the puckering atoms C14A [-0.270 (10) Å] and O4A [+0.231 (7) Å] from the mean plane of C1A/C2A/C15A/C16A; and puckering atoms C19A [-0.273 (9) Å] and O6A [+0.225 (7) Å] from the C6A/C7A/C21A/C22A plane, with the puckering parameters Q=0.415 (9)Å, θ=69.5 (12)° and φ=320.1 (14)° for C1A-C2A/C14A–C16A/O4A and Q=0.402 (9)Å, θ=62.6 (13)° and φ=316.4 (16)° for C6A-C7A/C19A–C21A/O6A rings in *A*. In molecule *B* the two chromene rings are in twisted boat conformations with the corresponding parameters of 0.421 (8)Å: 116.2 (11)°:142.7 (13)° and 0.368 (8)Å: 111.4 (14)°:140.3 (15)° respectively for the angular fashion C1B-C2B/C14B–C16B/O4B and the linear fashion C6B-C7B/C19B–C21B/O6B chromene rings with puckering atoms, C14B [+0.277 (8) Å], O4B [-0.250 (7) Å], C19B [+0.240 (9) Å] and O6B [-0.207 (7) Å]. Interestingly, the two chromene rings (angular and linear fashion chromenes) in *A* pucker in opposite directions and these puckering parameters are also opposite compared with those in *B* (Fig. 2) which is the cause that the two molecules *A* and *B* differ in their conformations.

Intramolecular O—H···O hydrogen bonds (Table 1) involving O3A and O3B hydroxy O atoms generate S(6) whereas the one involving O5A and O5B atoms generate S(5) ring motifs (Fig. 1) (Bernstein *et al.*, 1995). There are weak intramolecular C—H···O interactions in the crystal structure, [C16A—H16A···O2A and C16B—H16B···O2B], which generate two S(6) ring motifs. The bond distances in (I) are comparable to those in a related structure (Fun *et al.*, 2006).

The crystal packing of (I) is stabilized by weak C—H···O interactions (Table 1). The molecules are arranged into zig-zag chains along the *c* axis (Fig. 2) These chains are stacked along the *b* axis by π–π interactions with distance Cg_5_···Cg_20_ = 3.600 (5) Å (symmetry code: x, -1+y, z); Cg_5_ and Cg_20_ are the centroids of C5A–C8A/C12A-C13A and C5B–C8B/C12B–C13B rings, respectively. C—H···π interactions were also observed (Table 1).

### Experimental

The air-dried roots of *C. formosum* ssp. *pruniflorum* (5.00 kg) was extracted with CH_2_Cl_2_ (2 x 20 L, for a week) at room temperature and was further evaporated under reduced pressure to afford a deep green crude CH_2_Cl_2_ extract (58.87 g), which was subjected to QCC (Quick Column Chromatography) on silica gel using n-hexane as a first eluent and then increasing the polarity with acetone to give 12 fractions (F1-F12). Fractions F8-F11 were combined and separated by QCC eluting with 30% EtOAc-n-hexane to give 8 subfractions (F8A-F8H). Subfractions F8E and F8F were combined and then separated by QCC and eluted with 30% EtOAc-n-hexane to obtain 20 subfractions (F8E1-F8E20). Subfraction F8E10-F8E12 were combined and then separated by QCC and eluted with a gradient of CH_2_Cl_2_-n-hexane to give 12 subfractions (F8E10A-F8E10L). Subfraction FR8E10B was further purified by CC (Column Chromatography) and eluted with 5% acetone-n-hexane to give the title compound as yellow solid (4.5 mg). Yellow needle-shaped single crystals of the title compound suitable for *x*-ray structure determination were recrystallized from acetone/CH_3_OH (9.5:0.5, v/v) after several days (M.p. 478-480 K).

### Refinement

All H atoms were placed in calculated positions with d(O—H) = 0.82 Å and d(C—H) = 0.93 Å for aromatic and CH, and 0.96 Å for CH_3_ atoms. The *U*_iso_ values were constrained to be 1.5*U*_eq_ of the carrier atom for methyl H atoms and 1.2*U*_eq_ for the remaining H atoms. A rotating group model was used for the methyl groups. The highest residual electron density peak is located at 0.85 Å from O2A and the deepest hole is located at 0.76 Å from O1A. A total of 3445 Friedel pairs were merged before final refinement as there is no large anomalous dispersion for the determination of the absolute structure.

### Crystal data

**Table:**

C_23_H_20_O_6_	*F*(000) = 824
*M**_r_* = 392.39	*D*_x_ = 1.436 Mg m^−^^3^
Monoclinic, *P**c*	Melting point = 478–480 K
Hall symbol: P -2yc	Mo *K*α radiation, λ = 0.71073 Å
*a* = 7.5842 (3) Å	Cell parameters from 3554 reflections
*b* = 12.2937 (4) Å	θ = 2.0–26.0°
*c* = 19.6023 (6) Å	µ = 0.10 mm^−^^1^
β = 96.827 (2)°	*T* = 100 K
*V* = 1814.72 (11) Å^3^	Needle, yellow
*Z* = 4	0.37 × 0.13 × 0.07 mm

### Data collection

**Table:**

Bruker APEXII CCD area-detector diffractometer	3554 independent reflections
Radiation source: sealed tube	2954 reflections with *I* > 2σ(*I*)
graphite	*R*_int_ = 0.077
φ and ω scans	θ_max_ = 26.0°, θ_min_ = 2.0°
Absorption correction: multi-scan (*SADABS*; Bruker, 2005)	*h* = −9→9
*T*_min_ = 0.963, *T*_max_ = 0.993	*k* = −15→15
33718 measured reflections	*l* = −24→23

### Refinement

**Table:**

Refinement on *F*^2^	Primary atom site location: structure-invariant direct methods
Least-squares matrix: full	Secondary atom site location: difference Fourier map
*R*[*F*^2^ > 2σ(*F*^2^)] = 0.103	Hydrogen site location: inferred from neighbouring sites
*wR*(*F*^2^) = 0.270	H-atom parameters constrained
*S* = 1.04	*w* = 1/[σ^2^(*F*_o_^2^) + (0.1648*P*)^2^ + 5.5181*P*] where *P* = (*F*_o_^2^ + 2*F*_c_^2^)/3
3554 reflections	(Δ/σ)_max_ = 0.001
453 parameters	Δρ_max_ = 1.21 e Å^−^^3^
2 restraints	Δρ_min_ = −0.39 e Å^−^^3^

### Special details

**Table:**

Experimental. The crystal was placed in the cold stream of an Oxford Cryosystems Cobra open-flow nitrogen cryostat (Cosier & Glazer, 1986) operating at 100.0 (1) K.
Geometry. All esds (except the esd in the dihedral angle between two l.s. planes) are estimated using the full covariance matrix. The cell esds are taken into account individually in the estimation of esds in distances, angles and torsion angles; correlations between esds in cell parameters are only used when they are defined by crystal symmetry. An approximate (isotropic) treatment of cell esds is used for estimating esds involving l.s. planes.
Refinement. Refinement of F^2^ against ALL reflections. The weighted R-factor wR and goodness of fit S are based on F^2^, conventional R-factors R are based on F, with F set to zero for negative F^2^. The threshold expression of F^2^ > 2sigma(F^2^) is used only for calculating R-factors(gt) etc. and is not relevant to the choice of reflections for refinement. R-factors based on F^2^ are statistically about twice as large as those based on F, and R- factors based on ALL data will be even larger.

### Fractional atomic coordinates and isotropic or equivalent isotropic displacement parameters (Å^2^)

**Table:**

	*x*	*y*	*z*	*U*_iso_*/*U*_eq_	
O1A	0.2594 (8)	0.6225 (5)	0.5423 (3)	0.0284 (14)	
O2A	0.7864 (8)	0.5793 (5)	0.6095 (4)	0.0300 (15)	
O3A	0.7794 (8)	0.7513 (5)	0.6837 (3)	0.0299 (15)	
H3AA	0.8227	0.6975	0.6674	0.045*	
O4A	0.5559 (8)	0.2608 (5)	0.4390 (3)	0.0254 (13)	
O5A	0.2102 (8)	0.3007 (5)	0.4131 (3)	0.0289 (14)	
H5AA	0.2715	0.2500	0.4024	0.043*	
O6A	0.2246 (8)	0.9394 (5)	0.6739 (3)	0.0256 (14)	
C1A	0.6044 (13)	0.4207 (7)	0.5107 (5)	0.0251 (19)	
C2A	0.4941 (11)	0.3517 (7)	0.4674 (4)	0.0219 (6)	
C3A	0.3142 (12)	0.3725 (7)	0.4539 (4)	0.0252 (18)	
C4A	0.2407 (11)	0.4679 (7)	0.4795 (4)	0.0219 (6)	
H4AA	0.1211	0.4849	0.4685	0.026*	
C5A	0.2460 (11)	0.7814 (7)	0.6065 (5)	0.0254 (18)	
H5AB	0.1273	0.7895	0.5889	0.031*	
C6A	0.3293 (13)	0.8539 (7)	0.6537 (4)	0.0275 (14)	
C7A	0.5028 (12)	0.8473 (7)	0.6790 (5)	0.029 (2)	
C8A	0.6086 (12)	0.7602 (8)	0.6578 (5)	0.030 (2)	
C9A	0.6311 (12)	0.5885 (8)	0.5873 (4)	0.028 (2)	
C10A	0.5222 (12)	0.5135 (7)	0.5380 (5)	0.028 (2)	
C11A	0.3485 (13)	0.5325 (7)	0.5197 (5)	0.0271 (19)	
C12A	0.3513 (13)	0.6930 (8)	0.5859 (4)	0.0275 (14)	
C13A	0.5217 (13)	0.6810 (8)	0.6099 (5)	0.029 (2)	
C14A	0.7423 (11)	0.2633 (7)	0.4260 (5)	0.0219 (6)	
C15A	0.8475 (12)	0.3104 (7)	0.4909 (5)	0.0244 (13)	
H15A	0.9617	0.2847	0.5042	0.029*	
C16A	0.7838 (12)	0.3844 (7)	0.5280 (4)	0.0244 (13)	
H16A	0.8539	0.4139	0.5656	0.029*	
C17A	0.7541 (11)	0.3372 (7)	0.3641 (4)	0.0235 (17)	
H17A	0.6762	0.3105	0.3256	0.035*	
H17B	0.8739	0.3378	0.3529	0.035*	
H17C	0.7197	0.4097	0.3750	0.035*	
C18A	0.7952 (13)	0.1488 (7)	0.4115 (5)	0.031 (2)	
H18A	0.7115	0.1184	0.3760	0.047*	
H18B	0.7965	0.1059	0.4525	0.047*	
H18C	0.9115	0.1487	0.3968	0.047*	
C19A	0.2789 (11)	0.9818 (7)	0.7425 (4)	0.0219 (6)	
C20A	0.4757 (11)	0.9990 (8)	0.7499 (4)	0.0219 (6)	
H20A	0.5231	1.0607	0.7725	0.026*	
C21A	0.5832 (12)	0.9296 (7)	0.7253 (5)	0.0269 (19)	
H21A	0.7055	0.9332	0.7372	0.032*	
C22A	0.2221 (11)	0.9032 (7)	0.7950 (4)	0.0219 (6)	
H22A	0.2928	0.8383	0.7954	0.033*	
H22B	0.0992	0.8849	0.7832	0.033*	
H22C	0.2384	0.9363	0.8396	0.033*	
C23A	0.1815 (13)	1.0908 (8)	0.7424 (5)	0.030 (2)	
H23A	0.2025	1.1318	0.7025	0.046*	
H23B	0.2245	1.1310	0.7830	0.046*	
H23C	0.0564	1.0781	0.7417	0.046*	
O1B	0.7714 (6)	0.1279 (5)	0.6348 (3)	0.0170 (11)	
O2B	0.2460 (7)	0.1655 (5)	0.5601 (3)	0.0224 (13)	
O3B	0.2679 (7)	−0.0013 (5)	0.4835 (3)	0.0243 (14)	
H3BA	0.2190	0.0505	0.4997	0.036*	
O4B	0.4578 (7)	0.4885 (5)	0.7314 (3)	0.0234 (13)	
O5B	0.7974 (7)	0.4482 (5)	0.7649 (3)	0.0265 (14)	
H5BA	0.7316	0.4976	0.7742	0.032*	
O6B	0.8254 (8)	−0.1813 (5)	0.5014 (3)	0.0230 (13)	
C1B	0.4180 (11)	0.3280 (7)	0.6589 (4)	0.0219 (6)	
C2B	0.5194 (11)	0.3932 (7)	0.7039 (4)	0.0219 (6)	
C3B	0.7035 (11)	0.3754 (7)	0.7233 (4)	0.0195 (16)	
C4B	0.7810 (10)	0.2841 (7)	0.6989 (4)	0.0186 (16)	
H4BA	0.9004	0.2689	0.7120	0.022*	
C5B	0.7971 (10)	−0.0268 (6)	0.5681 (4)	0.0168 (9)	
H5BB	0.9150	−0.0335	0.5871	0.020*	
C6B	0.7222 (10)	−0.1000 (7)	0.5196 (4)	0.0168 (9)	
C7B	0.5435 (10)	−0.0911 (6)	0.4902 (4)	0.0177 (11)	
C8B	0.4416 (10)	−0.0059 (6)	0.5117 (4)	0.0139 (15)	
C9B	0.4054 (10)	0.1592 (6)	0.5834 (4)	0.0155 (15)	
C10B	0.4968 (9)	0.2335 (7)	0.6322 (4)	0.0158 (16)	
C11B	0.6794 (10)	0.2163 (7)	0.6551 (4)	0.0168 (9)	
C12B	0.6909 (11)	0.0571 (6)	0.5876 (4)	0.0177 (11)	
C13B	0.5122 (10)	0.0696 (7)	0.5609 (4)	0.0161 (16)	
C14B	0.2689 (10)	0.4816 (6)	0.7418 (4)	0.0145 (15)	
C15B	0.1709 (11)	0.4396 (7)	0.6785 (4)	0.0188 (16)	
H15B	0.0594	0.4685	0.6640	0.023*	
C16B	0.2338 (10)	0.3616 (7)	0.6407 (4)	0.0201 (17)	
H16B	0.1629	0.3299	0.6041	0.024*	
C17B	0.2476 (11)	0.4105 (7)	0.8039 (4)	0.0213 (13)	
H17D	0.3047	0.3417	0.7990	0.032*	
H17E	0.1236	0.3989	0.8069	0.032*	
H17F	0.3009	0.4459	0.8448	0.032*	
C18B	0.2195 (11)	0.6005 (7)	0.7543 (4)	0.0213 (13)	
H18D	0.2315	0.6426	0.7139	0.032*	
H18E	0.2972	0.6291	0.7923	0.032*	
H18F	0.0990	0.6040	0.7645	0.032*	
C19B	0.7833 (11)	−0.2314 (7)	0.4314 (4)	0.0216 (18)	
C20B	0.5868 (11)	−0.2446 (7)	0.4176 (5)	0.0244 (18)	
H20B	0.5415	−0.3022	0.3901	0.029*	
C21B	0.4738 (10)	−0.1771 (7)	0.4431 (5)	0.0240 (18)	
H21B	0.3520	−0.1846	0.4311	0.029*	
C22B	0.8534 (13)	−0.1520 (8)	0.3810 (5)	0.034 (2)	
H22D	0.9794	−0.1439	0.3921	0.050*	
H22E	0.7969	−0.0826	0.3842	0.050*	
H22F	0.8279	−0.1797	0.3351	0.050*	
C23B	0.8837 (12)	−0.3365 (7)	0.4352 (5)	0.028 (2)	
H23D	1.0078	−0.3223	0.4477	0.042*	
H23E	0.8670	−0.3718	0.3912	0.042*	
H23F	0.8408	−0.3828	0.4690	0.042*	

### Atomic displacement parameters (Å^2^)

**Table:**

	*U*^11^	*U*^22^	*U*^33^	*U*^12^	*U*^13^	*U*^23^
O1A	0.024 (3)	0.022 (3)	0.040 (4)	0.005 (2)	0.004 (3)	0.004 (3)
O2A	0.017 (3)	0.029 (3)	0.042 (4)	0.002 (3)	−0.002 (3)	0.007 (3)
O3A	0.018 (3)	0.037 (4)	0.034 (4)	0.013 (3)	0.001 (3)	−0.001 (3)
O4A	0.021 (3)	0.018 (3)	0.038 (4)	−0.006 (2)	0.005 (3)	0.002 (3)
O5A	0.023 (3)	0.025 (3)	0.037 (4)	−0.006 (2)	−0.002 (3)	−0.007 (3)
O6A	0.029 (3)	0.021 (3)	0.026 (3)	0.004 (3)	0.002 (3)	−0.002 (3)
C1A	0.043 (5)	0.012 (4)	0.023 (4)	−0.009 (4)	0.017 (4)	−0.003 (3)
C2A	0.0241 (14)	0.0209 (14)	0.0229 (14)	0.0040 (11)	0.0117 (11)	0.0043 (11)
C3A	0.035 (5)	0.020 (4)	0.021 (4)	−0.005 (3)	0.006 (4)	0.003 (3)
C4A	0.0241 (14)	0.0209 (14)	0.0229 (14)	0.0040 (11)	0.0117 (11)	0.0043 (11)
C5A	0.015 (4)	0.027 (4)	0.035 (5)	0.004 (3)	0.006 (3)	−0.002 (4)
C6A	0.046 (4)	0.025 (3)	0.013 (3)	−0.013 (3)	0.008 (3)	0.001 (2)
C7A	0.028 (5)	0.024 (4)	0.039 (5)	0.010 (4)	0.023 (4)	0.017 (4)
C8A	0.026 (4)	0.034 (5)	0.029 (5)	−0.018 (4)	0.004 (4)	0.002 (4)
C9A	0.028 (5)	0.035 (5)	0.019 (4)	−0.022 (4)	0.003 (4)	−0.001 (4)
C10A	0.035 (5)	0.017 (4)	0.036 (5)	0.008 (4)	0.027 (4)	0.015 (4)
C11A	0.037 (5)	0.023 (4)	0.023 (4)	−0.007 (4)	0.013 (4)	0.013 (3)
C12A	0.046 (4)	0.025 (3)	0.013 (3)	−0.013 (3)	0.008 (3)	0.001 (2)
C13A	0.033 (5)	0.023 (5)	0.032 (5)	0.003 (4)	0.011 (4)	0.005 (4)
C14A	0.0241 (14)	0.0209 (14)	0.0229 (14)	0.0040 (11)	0.0117 (11)	0.0043 (11)
C15A	0.034 (3)	0.019 (3)	0.021 (3)	−0.012 (2)	0.005 (2)	−0.001 (2)
C16A	0.034 (3)	0.019 (3)	0.021 (3)	−0.012 (2)	0.005 (2)	−0.001 (2)
C17A	0.021 (4)	0.029 (4)	0.021 (4)	−0.003 (3)	0.004 (3)	−0.002 (3)
C18A	0.043 (5)	0.022 (4)	0.030 (5)	0.010 (4)	0.011 (4)	−0.003 (4)
C19A	0.0241 (14)	0.0209 (14)	0.0229 (14)	0.0040 (11)	0.0117 (11)	0.0043 (11)
C20A	0.0241 (14)	0.0209 (14)	0.0229 (14)	0.0040 (11)	0.0117 (11)	0.0043 (11)
C21A	0.031 (5)	0.027 (5)	0.023 (4)	0.013 (4)	0.006 (3)	0.012 (4)
C22A	0.0241 (14)	0.0209 (14)	0.0229 (14)	0.0040 (11)	0.0117 (11)	0.0043 (11)
C23A	0.035 (5)	0.030 (5)	0.030 (5)	0.010 (4)	0.021 (4)	0.001 (4)
O1B	0.011 (2)	0.028 (3)	0.011 (3)	−0.001 (2)	0.001 (2)	−0.004 (2)
O2B	0.017 (3)	0.035 (3)	0.016 (3)	0.004 (2)	0.003 (2)	−0.003 (2)
O3B	0.015 (3)	0.033 (3)	0.023 (3)	0.005 (2)	−0.005 (2)	−0.007 (3)
O4B	0.017 (3)	0.018 (3)	0.036 (3)	0.007 (2)	0.010 (2)	−0.009 (3)
O5B	0.018 (3)	0.024 (3)	0.039 (4)	0.001 (2)	0.006 (3)	−0.009 (3)
O6B	0.028 (3)	0.017 (3)	0.023 (3)	0.004 (2)	−0.005 (2)	−0.010 (2)
C1B	0.0241 (14)	0.0209 (14)	0.0229 (14)	0.0040 (11)	0.0117 (11)	0.0043 (11)
C2B	0.0241 (14)	0.0209 (14)	0.0229 (14)	0.0040 (11)	0.0117 (11)	0.0043 (11)
C3B	0.023 (4)	0.022 (4)	0.015 (4)	0.000 (3)	0.007 (3)	−0.007 (3)
C4B	0.010 (3)	0.030 (4)	0.016 (4)	0.001 (3)	0.004 (3)	0.007 (3)
C5B	0.016 (2)	0.018 (2)	0.017 (2)	0.0005 (17)	0.0055 (17)	−0.0026 (18)
C6B	0.016 (2)	0.018 (2)	0.017 (2)	0.0005 (17)	0.0055 (17)	−0.0026 (18)
C7B	0.025 (3)	0.012 (2)	0.017 (3)	0.003 (2)	0.006 (2)	−0.006 (2)
C8B	0.018 (4)	0.009 (3)	0.016 (3)	0.000 (3)	0.008 (3)	0.004 (3)
C9B	0.018 (4)	0.013 (4)	0.016 (4)	0.005 (3)	0.004 (3)	0.005 (3)
C10B	0.012 (3)	0.026 (4)	0.009 (3)	0.000 (3)	0.000 (3)	−0.004 (3)
C11B	0.016 (2)	0.018 (2)	0.017 (2)	0.0005 (17)	0.0055 (17)	−0.0026 (18)
C12B	0.025 (3)	0.012 (2)	0.017 (3)	0.003 (2)	0.006 (2)	−0.006 (2)
C13B	0.016 (4)	0.026 (4)	0.006 (3)	0.001 (3)	−0.001 (3)	0.003 (3)
C14B	0.013 (3)	0.013 (4)	0.019 (4)	0.006 (3)	0.007 (3)	−0.001 (3)
C15B	0.024 (4)	0.015 (4)	0.016 (4)	0.008 (3)	0.000 (3)	−0.001 (3)
C16B	0.020 (4)	0.021 (4)	0.020 (4)	0.001 (3)	0.010 (3)	0.003 (3)
C17B	0.024 (3)	0.019 (3)	0.023 (3)	0.012 (2)	0.012 (2)	0.004 (2)
C18B	0.024 (3)	0.019 (3)	0.023 (3)	0.012 (2)	0.012 (2)	0.004 (2)
C19B	0.028 (4)	0.028 (4)	0.009 (3)	0.007 (3)	0.000 (3)	−0.008 (3)
C20B	0.023 (4)	0.016 (4)	0.031 (5)	0.002 (3)	−0.007 (3)	−0.006 (3)
C21B	0.009 (3)	0.031 (5)	0.029 (4)	−0.008 (3)	−0.007 (3)	−0.007 (4)
C22B	0.033 (5)	0.040 (5)	0.030 (5)	0.015 (4)	0.016 (4)	0.003 (4)
C23B	0.029 (4)	0.024 (4)	0.031 (5)	0.016 (4)	0.003 (4)	−0.006 (4)

### Geometric parameters (Å, °)

**Table:**

O1A—C12A	1.351 (11)	O1B—C12B	1.361 (9)
O1A—C11A	1.395 (11)	O1B—C11B	1.375 (9)
O2A—C9A	1.211 (11)	O2B—C9B	1.242 (9)
O3A—C8A	1.338 (11)	O3B—C8B	1.368 (9)
O3A—H3AA	0.8200	O3B—H3BA	0.8200
O4A—C2A	1.356 (10)	O4B—C2B	1.393 (10)
O4A—C14A	1.467 (10)	O4B—C14B	1.473 (9)
O5A—C3A	1.376 (10)	O5B—C3B	1.355 (10)
O5A—H5AA	0.8200	O5B—H5BA	0.8200
O6A—C6A	1.402 (11)	O6B—C6B	1.343 (9)
O6A—C19A	1.455 (11)	O6B—C19B	1.503 (9)
C1A—C2A	1.405 (13)	C1B—C2B	1.361 (13)
C1A—C16A	1.433 (14)	C1B—C10B	1.434 (11)
C1A—C10A	1.434 (12)	C1B—C16B	1.460 (11)
C2A—C3A	1.382 (12)	C2B—C3B	1.420 (12)
C3A—C4A	1.416 (12)	C3B—C4B	1.379 (11)
C4A—C11A	1.330 (13)	C4B—C11B	1.367 (12)
C4A—H4AA	0.9300	C4B—H4BA	0.9300
C5A—C6A	1.382 (12)	C5B—C6B	1.382 (11)
C5A—C12A	1.434 (12)	C5B—C12B	1.390 (10)
C5A—H5AB	0.9300	C5B—H5BB	0.9300
C6A—C7A	1.352 (14)	C6B—C7B	1.412 (11)
C7A—C8A	1.429 (13)	C7B—C8B	1.397 (10)
C7A—C21A	1.446 (14)	C7B—C21B	1.461 (11)
C8A—C13A	1.454 (13)	C8B—C13B	1.398 (11)
C9A—C13A	1.505 (13)	C9B—C10B	1.439 (11)
C9A—C10A	1.508 (13)	C9B—C13B	1.466 (10)
C10A—C11A	1.344 (14)	C10B—C11B	1.420 (10)
C12A—C13A	1.331 (14)	C12B—C13B	1.402 (11)
C14A—C18A	1.501 (12)	C14B—C15B	1.463 (11)
C14A—C17A	1.527 (12)	C14B—C17B	1.522 (11)
C14A—C15A	1.532 (12)	C14B—C18B	1.535 (10)
C15A—C16A	1.293 (13)	C15B—C16B	1.334 (11)
C15A—H15A	0.9300	C15B—H15B	0.9300
C16A—H16A	0.9300	C16B—H16B	0.9300
C17A—H17A	0.9600	C17B—H17D	0.9600
C17A—H17B	0.9600	C17B—H17E	0.9600
C17A—H17C	0.9600	C17B—H17F	0.9600
C18A—H18A	0.9600	C18B—H18D	0.9600
C18A—H18B	0.9600	C18B—H18E	0.9600
C18A—H18C	0.9600	C18B—H18F	0.9600
C19A—C20A	1.498 (11)	C19B—C20B	1.491 (11)
C19A—C22A	1.511 (11)	C19B—C23B	1.497 (11)
C19A—C23A	1.530 (12)	C19B—C22B	1.528 (13)
C20A—C21A	1.309 (12)	C20B—C21B	1.333 (12)
C20A—H20A	0.9300	C20B—H20B	0.9300
C21A—H21A	0.9300	C21B—H21B	0.9300
C22A—H22A	0.9600	C22B—H22D	0.9600
C22A—H22B	0.9600	C22B—H22E	0.9600
C22A—H22C	0.9600	C22B—H22F	0.9600
C23A—H23A	0.9600	C23B—H23D	0.9600
C23A—H23B	0.9600	C23B—H23E	0.9600
C23A—H23C	0.9600	C23B—H23F	0.9600
			
C12A—O1A—C11A	118.5 (7)	C12B—O1B—C11B	120.0 (6)
C8A—O3A—H3AA	109.5	C8B—O3B—H3BA	109.5
C2A—O4A—C14A	116.2 (6)	C2B—O4B—C14B	112.4 (6)
C3A—O5A—H5AA	109.5	C3B—O5B—H5BA	109.5
C6A—O6A—C19A	115.0 (7)	C6B—O6B—C19B	118.6 (6)
C2A—C1A—C16A	115.8 (8)	C2B—C1B—C10B	119.1 (8)
C2A—C1A—C10A	116.9 (9)	C2B—C1B—C16B	116.3 (8)
C16A—C1A—C10A	127.0 (9)	C10B—C1B—C16B	124.6 (8)
O4A—C2A—C3A	117.1 (8)	C1B—C2B—O4B	123.8 (8)
O4A—C2A—C1A	122.4 (8)	C1B—C2B—C3B	123.0 (8)
C3A—C2A—C1A	120.5 (8)	O4B—C2B—C3B	113.0 (7)
O5A—C3A—C2A	118.5 (8)	O5B—C3B—C4B	122.1 (7)
O5A—C3A—C4A	121.0 (8)	O5B—C3B—C2B	119.2 (7)
C2A—C3A—C4A	120.5 (8)	C4B—C3B—C2B	118.6 (8)
C11A—C4A—C3A	117.7 (8)	C11B—C4B—C3B	118.9 (7)
C11A—C4A—H4AA	121.2	C11B—C4B—H4BA	120.6
C3A—C4A—H4AA	121.2	C3B—C4B—H4BA	120.6
C6A—C5A—C12A	116.8 (8)	C6B—C5B—C12B	117.9 (7)
C6A—C5A—H5AB	121.6	C6B—C5B—H5BB	121.1
C12A—C5A—H5AB	121.6	C12B—C5B—H5BB	121.1
C7A—C6A—C5A	123.6 (9)	O6B—C6B—C5B	117.5 (7)
C7A—C6A—O6A	120.0 (8)	O6B—C6B—C7B	120.7 (7)
C5A—C6A—O6A	116.3 (8)	C5B—C6B—C7B	121.8 (7)
C6A—C7A—C8A	119.6 (9)	C8B—C7B—C6B	118.3 (7)
C6A—C7A—C21A	120.5 (8)	C8B—C7B—C21B	124.2 (7)
C8A—C7A—C21A	119.9 (8)	C6B—C7B—C21B	117.3 (7)
O3A—C8A—C7A	120.2 (8)	O3B—C8B—C7B	116.7 (7)
O3A—C8A—C13A	122.2 (9)	O3B—C8B—C13B	121.5 (6)
C7A—C8A—C13A	117.5 (9)	C7B—C8B—C13B	121.7 (7)
O2A—C9A—C13A	120.7 (8)	O2B—C9B—C10B	125.2 (7)
O2A—C9A—C10A	127.5 (9)	O2B—C9B—C13B	118.9 (7)
C13A—C9A—C10A	111.8 (8)	C10B—C9B—C13B	115.9 (7)
C11A—C10A—C1A	119.7 (10)	C11B—C10B—C1B	116.0 (7)
C11A—C10A—C9A	120.6 (8)	C11B—C10B—C9B	119.4 (7)
C1A—C10A—C9A	119.7 (8)	C1B—C10B—C9B	124.6 (7)
C4A—C11A—C10A	124.6 (9)	C4B—C11B—O1B	113.3 (7)
C4A—C11A—O1A	112.0 (8)	C4B—C11B—C10B	124.3 (7)
C10A—C11A—O1A	123.3 (9)	O1B—C11B—C10B	122.4 (7)
C13A—C12A—O1A	124.0 (9)	O1B—C12B—C5B	115.7 (7)
C13A—C12A—C5A	122.3 (8)	O1B—C12B—C13B	121.2 (7)
O1A—C12A—C5A	113.7 (8)	C5B—C12B—C13B	123.0 (7)
C12A—C13A—C8A	120.0 (9)	C8B—C13B—C12B	117.3 (7)
C12A—C13A—C9A	121.7 (9)	C8B—C13B—C9B	121.7 (7)
C8A—C13A—C9A	118.2 (8)	C12B—C13B—C9B	121.0 (7)
O4A—C14A—C18A	107.3 (7)	C15B—C14B—O4B	107.9 (6)
O4A—C14A—C17A	107.4 (7)	C15B—C14B—C17B	112.5 (7)
C18A—C14A—C17A	111.4 (7)	O4B—C14B—C17B	110.0 (6)
O4A—C14A—C15A	106.3 (7)	C15B—C14B—C18B	111.2 (7)
C18A—C14A—C15A	112.8 (8)	O4B—C14B—C18B	103.1 (6)
C17A—C14A—C15A	111.2 (7)	C17B—C14B—C18B	111.6 (6)
C16A—C15A—C14A	122.7 (9)	C16B—C15B—C14B	123.1 (7)
C16A—C15A—H15A	118.7	C16B—C15B—H15B	118.5
C14A—C15A—H15A	118.7	C14B—C15B—H15B	118.5
C15A—C16A—C1A	119.6 (8)	C15B—C16B—C1B	117.5 (8)
C15A—C16A—H16A	120.2	C15B—C16B—H16B	121.2
C1A—C16A—H16A	120.2	C1B—C16B—H16B	121.2
C14A—C17A—H17A	109.5	C14B—C17B—H17D	109.5
C14A—C17A—H17B	109.5	C14B—C17B—H17E	109.5
H17A—C17A—H17B	109.5	H17D—C17B—H17E	109.5
C14A—C17A—H17C	109.5	C14B—C17B—H17F	109.5
H17A—C17A—H17C	109.5	H17D—C17B—H17F	109.5
H17B—C17A—H17C	109.5	H17E—C17B—H17F	109.5
C14A—C18A—H18A	109.5	C14B—C18B—H18D	109.5
C14A—C18A—H18B	109.5	C14B—C18B—H18E	109.5
H18A—C18A—H18B	109.5	H18D—C18B—H18E	109.5
C14A—C18A—H18C	109.5	C14B—C18B—H18F	109.5
H18A—C18A—H18C	109.5	H18D—C18B—H18F	109.5
H18B—C18A—H18C	109.5	H18E—C18B—H18F	109.5
O6A—C19A—C20A	108.0 (6)	C20B—C19B—C23B	114.0 (8)
O6A—C19A—C22A	109.1 (7)	C20B—C19B—O6B	108.1 (6)
C20A—C19A—C22A	112.7 (7)	C23B—C19B—O6B	104.9 (7)
O6A—C19A—C23A	103.3 (7)	C20B—C19B—C22B	111.7 (7)
C20A—C19A—C23A	110.6 (8)	C23B—C19B—C22B	111.6 (7)
C22A—C19A—C23A	112.6 (7)	O6B—C19B—C22B	106.0 (7)
C21A—C20A—C19A	122.0 (9)	C21B—C20B—C19B	122.5 (7)
C21A—C20A—H20A	119.0	C21B—C20B—H20B	118.8
C19A—C20A—H20A	119.0	C19B—C20B—H20B	118.8
C20A—C21A—C7A	116.9 (9)	C20B—C21B—C7B	119.2 (7)
C20A—C21A—H21A	121.6	C20B—C21B—H21B	120.4
C7A—C21A—H21A	121.6	C7B—C21B—H21B	120.4
C19A—C22A—H22A	109.5	C19B—C22B—H22D	109.5
C19A—C22A—H22B	109.5	C19B—C22B—H22E	109.5
H22A—C22A—H22B	109.5	H22D—C22B—H22E	109.5
C19A—C22A—H22C	109.5	C19B—C22B—H22F	109.5
H22A—C22A—H22C	109.5	H22D—C22B—H22F	109.5
H22B—C22A—H22C	109.5	H22E—C22B—H22F	109.5
C19A—C23A—H23A	109.5	C19B—C23B—H23D	109.5
C19A—C23A—H23B	109.5	C19B—C23B—H23E	109.5
H23A—C23A—H23B	109.5	H23D—C23B—H23E	109.5
C19A—C23A—H23C	109.5	C19B—C23B—H23F	109.5
H23A—C23A—H23C	109.5	H23D—C23B—H23F	109.5
H23B—C23A—H23C	109.5	H23E—C23B—H23F	109.5
			
C14A—O4A—C2A—C3A	153.0 (7)	C10B—C1B—C2B—O4B	178.1 (7)
C14A—O4A—C2A—C1A	−29.3 (11)	C16B—C1B—C2B—O4B	−1.1 (12)
C16A—C1A—C2A—O4A	−5.1 (12)	C10B—C1B—C2B—C3B	3.3 (12)
C10A—C1A—C2A—O4A	−179.3 (7)	C16B—C1B—C2B—C3B	−175.9 (7)
C16A—C1A—C2A—C3A	172.5 (7)	C14B—O4B—C2B—C1B	33.4 (11)
C10A—C1A—C2A—C3A	−1.7 (12)	C14B—O4B—C2B—C3B	−151.3 (7)
O4A—C2A—C3A—O5A	0.0 (11)	C1B—C2B—C3B—O5B	175.7 (7)
C1A—C2A—C3A—O5A	−177.7 (7)	O4B—C2B—C3B—O5B	0.4 (11)
O4A—C2A—C3A—C4A	−177.6 (7)	C1B—C2B—C3B—C4B	−4.2 (12)
C1A—C2A—C3A—C4A	4.7 (12)	O4B—C2B—C3B—C4B	−179.5 (7)
O5A—C3A—C4A—C11A	178.7 (7)	O5B—C3B—C4B—C11B	−177.6 (7)
C2A—C3A—C4A—C11A	−3.8 (12)	C2B—C3B—C4B—C11B	2.3 (12)
C12A—C5A—C6A—C7A	2.1 (13)	C19B—O6B—C6B—C5B	−153.9 (7)
C12A—C5A—C6A—O6A	−179.4 (7)	C19B—O6B—C6B—C7B	26.5 (11)
C19A—O6A—C6A—C7A	−29.2 (11)	C12B—C5B—C6B—O6B	−179.6 (7)
C19A—O6A—C6A—C5A	152.2 (7)	C12B—C5B—C6B—C7B	0.0 (11)
C5A—C6A—C7A—C8A	−1.2 (13)	O6B—C6B—C7B—C8B	179.1 (7)
O6A—C6A—C7A—C8A	−179.6 (7)	C5B—C6B—C7B—C8B	−0.5 (12)
C5A—C6A—C7A—C21A	176.5 (8)	O6B—C6B—C7B—C21B	3.9 (11)
O6A—C6A—C7A—C21A	−1.9 (12)	C5B—C6B—C7B—C21B	−175.7 (7)
C6A—C7A—C8A—O3A	−178.4 (8)	C6B—C7B—C8B—O3B	−178.4 (7)
C21A—C7A—C8A—O3A	3.8 (12)	C21B—C7B—C8B—O3B	−3.5 (11)
C6A—C7A—C8A—C13A	−0.9 (12)	C6B—C7B—C8B—C13B	0.3 (11)
C21A—C7A—C8A—C13A	−178.7 (7)	C21B—C7B—C8B—C13B	175.1 (7)
C2A—C1A—C10A—C11A	−2.1 (12)	C2B—C1B—C10B—C11B	−0.7 (11)
C16A—C1A—C10A—C11A	−175.6 (8)	C16B—C1B—C10B—C11B	178.5 (7)
C2A—C1A—C10A—C9A	177.4 (7)	C2B—C1B—C10B—C9B	−178.7 (7)
C16A—C1A—C10A—C9A	4.0 (13)	C16B—C1B—C10B—C9B	0.4 (13)
O2A—C9A—C10A—C11A	177.6 (9)	O2B—C9B—C10B—C11B	179.5 (7)
C13A—C9A—C10A—C11A	−0.6 (11)	C13B—C9B—C10B—C11B	0.0 (10)
O2A—C9A—C10A—C1A	−1.9 (13)	O2B—C9B—C10B—C1B	−2.5 (13)
C13A—C9A—C10A—C1A	179.8 (7)	C13B—C9B—C10B—C1B	178.0 (7)
C3A—C4A—C11A—C10A	−0.2 (13)	C3B—C4B—C11B—O1B	−179.6 (7)
C3A—C4A—C11A—O1A	−179.0 (7)	C3B—C4B—C11B—C10B	0.3 (12)
C1A—C10A—C11A—C4A	3.1 (13)	C12B—O1B—C11B—C4B	−175.6 (7)
C9A—C10A—C11A—C4A	−176.4 (8)	C12B—O1B—C11B—C10B	4.6 (11)
C1A—C10A—C11A—O1A	−178.1 (7)	C1B—C10B—C11B—C4B	−1.1 (12)
C9A—C10A—C11A—O1A	2.3 (12)	C9B—C10B—C11B—C4B	177.0 (7)
C12A—O1A—C11A—C4A	177.5 (7)	C1B—C10B—C11B—O1B	178.7 (7)
C12A—O1A—C11A—C10A	−1.4 (12)	C9B—C10B—C11B—O1B	−3.2 (11)
C11A—O1A—C12A—C13A	−1.4 (12)	C11B—O1B—C12B—C5B	177.2 (6)
C11A—O1A—C12A—C5A	−179.1 (7)	C11B—O1B—C12B—C13B	−2.7 (11)
C6A—C5A—C12A—C13A	−0.8 (13)	C6B—C5B—C12B—O1B	−179.2 (7)
C6A—C5A—C12A—O1A	177.0 (7)	C6B—C5B—C12B—C13B	0.8 (12)
O1A—C12A—C13A—C8A	−178.8 (8)	O3B—C8B—C13B—C12B	179.0 (7)
C5A—C12A—C13A—C8A	−1.3 (13)	C7B—C8B—C13B—C12B	0.5 (11)
O1A—C12A—C13A—C9A	3.1 (14)	O3B—C8B—C13B—C9B	−1.6 (11)
C5A—C12A—C13A—C9A	−179.3 (7)	C7B—C8B—C13B—C9B	179.8 (7)
O3A—C8A—C13A—C12A	179.6 (8)	O1B—C12B—C13B—C8B	178.9 (7)
C7A—C8A—C13A—C12A	2.2 (13)	C5B—C12B—C13B—C8B	−1.0 (11)
O3A—C8A—C13A—C9A	−2.3 (13)	O1B—C12B—C13B—C9B	−0.5 (11)
C7A—C8A—C13A—C9A	−179.7 (7)	C5B—C12B—C13B—C9B	179.6 (7)
O2A—C9A—C13A—C12A	179.6 (8)	O2B—C9B—C13B—C8B	2.9 (11)
C10A—C9A—C13A—C12A	−2.0 (12)	C10B—C9B—C13B—C8B	−177.6 (7)
O2A—C9A—C13A—C8A	1.5 (12)	O2B—C9B—C13B—C12B	−177.7 (7)
C10A—C9A—C13A—C8A	179.9 (7)	C10B—C9B—C13B—C12B	1.8 (11)
C2A—O4A—C14A—C18A	166.4 (7)	C2B—O4B—C14B—C15B	−48.5 (9)
C2A—O4A—C14A—C17A	−73.7 (9)	C2B—O4B—C14B—C17B	74.5 (8)
C2A—O4A—C14A—C15A	45.5 (9)	C2B—O4B—C14B—C18B	−166.3 (7)
O4A—C14A—C15A—C16A	−34.4 (11)	O4B—C14B—C15B—C16B	38.8 (11)
C18A—C14A—C15A—C16A	−151.7 (8)	C17B—C14B—C15B—C16B	−82.7 (10)
C17A—C14A—C15A—C16A	82.2 (10)	C18B—C14B—C15B—C16B	151.2 (8)
C14A—C15A—C16A—C1A	3.6 (13)	C14B—C15B—C16B—C1B	−8.3 (12)
C2A—C1A—C16A—C15A	18.1 (12)	C2B—C1B—C16B—C15B	−12.5 (11)
C10A—C1A—C16A—C15A	−168.4 (8)	C10B—C1B—C16B—C15B	168.3 (8)
C6A—O6A—C19A—C20A	46.9 (9)	C6B—O6B—C19B—C20B	−41.9 (10)
C6A—O6A—C19A—C22A	−75.9 (8)	C6B—O6B—C19B—C23B	−163.9 (7)
C6A—O6A—C19A—C23A	164.1 (7)	C6B—O6B—C19B—C22B	77.9 (8)
O6A—C19A—C20A—C21A	−40.2 (11)	C23B—C19B—C20B—C21B	147.1 (9)
C22A—C19A—C20A—C21A	80.4 (10)	O6B—C19B—C20B—C21B	30.9 (12)
C23A—C19A—C20A—C21A	−152.6 (8)	C22B—C19B—C20B—C21B	−85.3 (11)
C19A—C20A—C21A—C7A	12.1 (12)	C19B—C20B—C21B—C7B	−4.3 (14)
C6A—C7A—C21A—C20A	10.9 (12)	C8B—C7B—C21B—C20B	169.7 (8)
C8A—C7A—C21A—C20A	−171.4 (8)	C6B—C7B—C21B—C20B	−15.4 (12)

### Hydrogen-bond geometry (Å, °)

**Table:**

Cg5 and Cg20 are the centroids of C5A–C8A/C12A/C13A and C5B–C8B/C12B/C13B rings, respectively.

**Table:**

*D*—H···*A*	*D*—H	H···*A*	*D*···*A*	*D*—H···*A*
O3A—H3AA···O2A	0.82	1.84	2.571 (9)	147
O5A—H5AA···O4A	0.82	2.19	2.656 (9)	116
O3B—H3BA···O2B	0.82	1.84	2.559 (9)	146
O5B—H5BA···O4B	0.82	2.15	2.628 (8)	117
C15B—H15B···O2A^i^	0.93	2.60	3.514 (11)	168
C16A—H16A···O2A	0.93	2.29	2.879 (11)	121
C16B—H16B···O2B	0.93	2.31	2.890 (10)	120
C17B—H17E···O5B^i^	0.96	2.58	3.441 (10)	149
C23B—H23D···O1A^ii^	0.96	2.59	3.370 (11)	139
C18A—H18B···Cg20	0.96	2.75	3.611 (10)	150
C18B—H18D···Cg5	0.96	2.79	3.662 (9)	152

Symmetry codes:  (i) *x*−1, *y*, *z*; (ii) *x*+1, *y*−1, *z*.
